# Parietal Lobes in Schizophrenia: Do They Matter?

**DOI:** 10.1155/2011/581686

**Published:** 2011-10-16

**Authors:** Murat Yildiz, Stefan J. Borgwardt, Gregor E. Berger

**Affiliations:** ^1^Department of Neurology, Cantonal Hospital St. Gallen, 9007 St. Gallen, Switzerland; ^2^Psychiatric Outpatient Clinic, University Hospital Basel, 4031 Basel, Switzerland; ^3^Integrierte Psychiatrie Winterthur, Zurich Unterland, 8408 Winterthur, Switzerland

## Abstract

*Objective*. Despite observations that abnormal parietal lobe (PL) function is associated with psychotic-like experiences, our knowledge about the nature of PL involvement in schizophrenia is modest. The objective of this paper is to investigate the role of the PL in schizophrenia. *Method*. Medline databases were searched for English language publications using the following key words: parietal lobe, combined with schizophrenia, lesions, epilepsy, cognition, rare genetic disorders, MRI, fMRI, PET, and SPECT, respectively, followed by cross-checking of references. *Results*. Imaging studies in childhood onset schizophrenia suggest that grey matter abnormalities start in parietal and occipital lobes and proceed to frontal regions. Although, the findings are inconsistent, several studies with patients at risk to develop schizophrenia indicate early changes in the PL. *Conclusions*. We want to propose that in a proportion of individuals with emerging schizophrenia structural and functional alterations may start in the PL and progress to frontal regions.

## 1. Introduction

Despite observations that abnormal PL function is associated with psychotic-like experiences, our knowledge about the nature of PL involvement in schizophrenia (SZ) is relatively modest [1]. The PL is engaged in various neuropsychological functions which are affected in schizophrenic patients [2]. The PL supports the frontal lobe in storage and retrieval of verbal information [3]. Episodic memory encoding depends not only on recruiting prefrontal and medial temporal lobes but also on the activation of PL subregions [4]. The right inferior and medial parietal cortices have been associated with the ability to remember past events and arrange them in the right chronological order, consequently enabling us to conceive actions as controlled by ourselves and not a third person [5]. Finally, substantial processing for spatial perception, attention, and self-awareness takes place in the parietal lobes [6–8]. 

The PL forms strong anatomical connections with the frontal lobe [9]. Frontoparietal white matter maturation correlates with an increase in grey matter activity in both lobes during performance of a working memory (WM) task indicating the close functional connection [10]. Both lobes are frequently activated together while performing cognitive tasks [11]. Joint activation is characterised by a partially symmetrical anteroposterior ordering of activations in both lobes [12]. Croizé et al. demonstrated that although the frontoparietal network is activated in working memory (WM) tasks, both lobes perform distinctive processes [13]. 

First, we examine the neuropsychiatric manifestations of PL lesions, following a review of selected studies investigating the PL's contribution to SZ. The reevaluation of recent findings allows us to better understand the scope of the PL's influence in SZ. Finally, we propose a new disease model for SZ. A speculative hypothesis will be discussed suggesting that the timing and location of PL changes may differentiate one major pathway in the emergence and progression of SZ.

## 2. Method

Medline databases were searched for English language publications dating from 1966 to February 2011 using the following key words: parietal lobe combined with each of the following key words: schizophrenia, lesions, epilepsy, cognition, rare genetic disorders, MRI, fMRI, PET, and SPECT. Cross-checking of references led to the identification of additional relevant references.

### 2.1. The Anatomical Structure

The parietal lobe can be divided into 3 subregions: the postcentral gyrus, the superior parietal gyrus, and the inferior parietal lobule, consisting of two distinct areas: the angular gyrus and the supramarginal gyrus. The postcentral sulcus separates the postcentral gyrus from the superior parietal gyrus and the inferior parietal lobule. The posteromedial part of the parietal lobe is called the precuneus. The boundaries of the medial surface of the parietal lobe are the frontal, occipital lobes, and the cingulate gyrus. The central sulcus separates the frontal from the parietal lobe. The parieto-occipital fissure lies between the parietal and occipital lobes, and the subparietal sulcus lies between the parietal lobe and cingulate gyrus. From the temporal lobe, the parietal lobe is separated anteriorly by the Sylvian fissure [37]. 

### 2.2. Parietal Lobe Lesions and Their Neuropsychiatric Manifestations

#### 2.2.1. Stroke and Other Vascular Conditions

Lesions in the PL offer a unique opportunity to identify the consequences of altered PL function (see Table 1). In schizophrenic patients, dysfunctions can be seen which resemble symptoms seen in patients with PL lesions. Right PL injuries are often resulting in abnormal behaviour such as anosognosia [65]. This condition is associated with the denial of any impairment in the face of a hemiparetic limb [66, 67]. Unawareness of disease, lack of insight, is a phenomenon frequently seen in schizophrenic patients [68, 69]. Unawareness in SZ might occur in connection with deficits of executive functions [70] and thus be very similar to the concept of anosognosia in patients with detectable damage in the PL [71]. An association between structural damage in the frontal lobe and poor insight into illness could be established in schizophrenics [72]. The role of the PL in the phenomenon of unawareness in SZ has yet to be investigated in more detail in the context of the frontoparietal network.

A more obscure neuropsychiatric manifestations is alien hand syndrome (AHS) that may occur after damage of the PL as well as in other brain regions such as the frontal lobe or the corpus callosum [73]. The core symptom of AHS is the perceived loss of control over one's own body movements, that is, a limb. Patients suffer from the impression that an external force is responsible for movements of their own limbs; this symptom is seen occasionally in SZ as well [72]. Sporadically, patients with left PL ischemic lesions display ideomotor apraxia [73, 74] and have difficulties differentiating self-generated movements from foreign made movements [21]. Similar findings could be reproduced in healthy participants using transcranial magnetic stimulation of the superior parietal lobule [75]. These findings best correspond to passivity phenomena in SZ, which were associated with PL dysfunctions [76]. These patients report alien control of their limbs due to their inability to align the timing of motor actions internally [77]. 

Lesions in the posterior parietal cortex highlight the role of this region in disengaging attention from the current focus to a new one [78]. A deterioration of the ability to shift attention has been shown by several research groups in SZ [79]. Additionally, patients with PL lesions may experience difficulties to direct attention to the contralateral exterior world probably due to a disconnection of parieto-frontal networks [15, 80]. A lateralised defect in the control of attention was associated with the severity of symptoms in SZ [81]. Direct comparisons of the performances of schizophrenics and patients with neglect reveal qualitatively similar impairments [82]. Though, in some schizophrenics defects in spatial [83] and temporal perception are not as impressive as neglect seen in patients with PL lesions [84] and may be the reason that it has not attracted much attention so far.

#### 2.2.2. Parietal Lobe Epilepsy

Focal epilepsy affecting the parietal lobe may also present itself with psychotic symptoms. In a retrospective Canadian study of patients with parietal lobe epilepsy, most patients experienced aurae, nearly all being somatosensory. Some patients described disturbances of body image, visual illusions, vertiginous sensations, and aphasia. A few patients expressed complex visual or auditory hallucinations [85]. Parietal lobe epilepsy often cooccurs with temporal lobe epilepsy which is often accompanied with psychotic like experiences. Marsh et al. described that grey matter volume in the temporal lobes and frontoparietal regions was significantly smaller not only in patients with epilepsy and chronic interictal psychosis but also in patients with unilateral temporal lobe epilepsy without chronic psychosis compared with healthy control subjects [86].

#### 2.2.3. Rare Genetic Disorders with Parietal Involvement

Velocardiofacial (VCF) syndrome is associated with aberrant parietal and frontotemporal white matter tracts. The VCF syndrome is a rare genetic disorder with a prevalence estimated at 1 in 4,000 live births caused by deletion in chromosome 22q11.2 and characterized by cardiac and facial abnormalities. 25% of VCF cases present SZ-like symptoms [87] and visuospatial cognitive impairments in adulthood associated with posterior parietal abnormalities [88]. In VCF, autism spectrum disorders [79] and obsessive-compulsive disorder [89] occur frequently. Another rare genetic disorder, fragile X syndrome, is associated with impaired visual motion processing involving primarily the PL [90]. Mental retardation is the hallmark of fragile X syndrome caused by silencing of the fragile-X mental retardation (fraX) gene [91]. Interestingly, Rivera et al. demonstrated that the parietal region was more active in patients having a higher expression of fraX gene [92].

### 2.3. Parietal Lobes in SZ

#### 2.3.1. Structural Imaging Studies with Parietal Involvement in Childhood-Onset Schizophrenia

Childhood onset schizophrenia (COS) is defined by its younger age of onset compared to its adult equivalent and may be a more homogeneous group [93]. Studies in COS are particularly important as they are supposed to be more genetically determined than the adult onset form. As seen in the case of very early onset SZ more severe premorbid neurodevelopmental abnormalities, a higher rate of cytogenetic anomalies and familial SZ are observed than in later onset cases [42]. 

Thompson et al. described that a dynamic wave of grey matter loss occurs, beginning in the PL and proceeding to the temporal and finally to the prefrontal dorsolateral cortices [94, 95]. The latter findings suggest that the changes in the PL occur early on in the disease. Although serial brain MRI scans in healthy children over a ten-year period revealed a similar pattern of grey-matter loss beginning in the dorsal parietal and primary sensorimotor regions spreading laterally and caudally into temporal cortices and anteriorly into dorsolateral prefrontal areas [32]. The pattern matches the order of grey matter loss seen in *COS; *however, the extend of loss is larger in COS. Additionally, Kyriakopoulos et al. demonstrated that compared with healthy controls, individuals with adolescent-onset SZ showed fractional anisotropy decrease in parietal regions, in contrast to individuals with adult onset SZ who showed additional fractional anisotropy reductions in frontal and temporal regions [96]. Fractional anisotropy is measured with diffusion tensor imaging and is positively correlated with the degree of neuronal maturation and organisation of white matter tracts [97]. These findings support the concept of SZ as a neurodegenerative disease [29].

#### 2.3.2. Structural Imaging Studies with Parietal Involvement in High-Risk Patients

In a longitudinal study over two years with a genetically defined high-risk (GHR) cohort from Edinburgh a significant decline in grey matter density was found in the right parietal, right frontal, and temporal lobes [30]. In another study, patients at ultra-high risk (UHR) experiencing prodromal symptoms showed significant cortical thinning in the inferior parietal cortex compared to healthy controls [98]. Borgwardt et al. observed that UHR subjects who later developed psychosis (converters) showed volume reductions in the medial and superior parietal, in the frontal and inferior temporal cortex and in the cerebellum. There were no longitudinal volumetric changes in UHR subjects who did not develop psychosis (nonconverters) [99]. These findings suggest that PL changes may occur prior to the first psychotic episode. However, a previous study from Pantelis et al. could not trace over one year significant changes in the PL of converters. Instead, they demonstrated that converters compared to nonconverters show a longitudinal reduction in the grey matter of the right medial temporal, lateral temporal, inferior frontal, and cingulate cortex bilaterally [39].

#### 2.3.3. Structural Imaging Studies with Parietal Involvement in First-Episode Psychosis and Established Schizophrenia

Reduced cortical thickness in the parietal and frontal regions is already evident in first-episode SZ patients (FE) [40]. It should be noted that a longitudinal surface contraction in frontal and parietal regions of the cortex was found in FE, but not in chronic schizophrenics [33, 100]. Prefrontal and temporoparietal grey matter volume reductions correlate significantly with cognitive performance in FE, indicating the clinical importance of such alterations [84]. 

Converging evidence suggests that chronic schizophrenics display PL structural abnormalities (see Table 2). Rowland et al. demonstrated with diffusion tensor imaging (DTI) a declined fractional anisotropy of white matter tracts connecting frontal and parietal regions in schizophrenics with negative symptoms [34]. Intriguingly, in the longitudinal four-year followup conducted by Mitelman et al., grey matter volumes in the parietal, frontal, and temporal lobes in schizophrenics with a poor clinical outcome continued to decline more rapidly compared to patients with a good clinical outcome [101]. Using three-dimensional cortical surface maps, a comparison between schizophrenics compared to their unaffected monozygotic cotwins revealed deficits in the superior parietal lobe, dorsolateral prefrontal cortex, and superior temporal gyrus [102]. Additionally, a previous study by the same research group demonstrated that frontal lobe grey matter deficits were present in affected and nonaffected twins [48]. 

### 2.4. Functional Parietal Lobe Abnormalities

#### 2.4.1. Functional Imaging Studies with Parietal Involvement in at Risk Mental States

Functional MRI (fMRI) studies indicate that UHR individuals display abnormal activation in the prefrontal and parietal cortex during performance of WM tasks [63]. Whalley et al. reported promising results from a recent longitudinal fMRI study in GHR individuals. fMRI data of converters compared to nonconverters showed increased activation of the PL and decreased activation of the anterior cingulate. However, the PL activation only had high predictive power if the lingual gyrus was also activated [64]. The study was limited as only 4 out of 62 at risk individuals developed SZ. Nevertheless, this study might demonstrate that parietal functional abnormalities are present in high-risk subjects who later become psychotic. 

The same study group found in GHR individuals increased connectivity between the left parietal and left prefrontal regions compared to healthy controls. The authors interpreted the hyperactivation of the PL as compensatory, since there were no differences in performance between the groups [64]. These results underline that many PL findings are reported in connection with the frontoparietal network.

#### 2.4.2. Functional Imaging Studies with Parietal Involvement in First-Episode Psychosis and in Chronic Schizophrenia

Prefrontal cortex dysfunctions have been identified as a key factor in SZ [103]. The role of PL dysfunctions still remains ambiguous in SZ. Parietal hypoactivation and ventrolateral prefrontal hyperactivity during WM tasks in FE patients indicate that frontoparietal networks are impaired early in the course of the illness [60]. Correspondingly, in patients with chronic SZ, parietal and frontal cortex activation deficits were described in WM tasks [104]. There is an increasing body of studies associating PL dysfunctions with a variety of symptoms in chronic SZ [105]. For instance, a PET study correlated positively regional cerebral blood flow in right superior parietal cortex with the severity of Schneiderian first-rank symptoms (voices conversing or commenting; thought broadcasting, withdrawal or insertion; made actions and thoughts) [49]. Moreover, Menon et al. demonstrated that thinking disturbance was correlated with deficits in activation in the parietal and the right frontal cortices [104]. In fact, overactivity in the right inferior parietal cortex was associated with the presence of delusions of control in a study with acute psychotic schizophrenics performing movement tasks. Interestingly, the activity in this region returned to normal levels when patients were symptom-free [1]. Lower levels of activation during verbal WM task performance in the left hemisphere across frontal and parietal regions were associated with poorer role functioning and greater severity of negative and disorganised symptoms [59]. A recent study identified disturbed parieto-occipital functional connectivity as related with positive symptoms of SZ [50]. These findings underline that two core symptoms of SZ, cognitive deficits and delusions, may be related to malfunctions in the parietal lobe [106].

## 3. Discussion

The nature of the pathological processes underlying progressive structural and functional changes in SZ and their exact timing in the brains remains unclear. Our paper provides a selection of studies indicating that PL lesions and epilepsy may cause psychotic-like symptoms and supports the concept that PL abnormalities could be important for SZ and related disorders. Structural brain irregularities in PL were found in imaging studies in COS suggesting that grey matter abnormalities start in parietal and occipital lobes and proceed in a dynamic wave to frontal cortices [42, 94]. This illustrates that PL structural alterations may occur early in the course of illness and points to genetic influences, yet to be determined [93]. The contribution of genetic factors to parietal involvement in SZ is further supported by a significant decline in grey matter found over time in GHR subjects [29]. The brain-derived neurotrophic factor (BDNF) could be one of the candidate genes for parietal lobe alterations seen in SZ. Indeed, there was decreased activity in the bilateral posterior parietal regions in GHR patients with BDNF Val homozygote versus BDNF Met carriers [107]. 

There is evidence that parietal lobe abnormalities may be only partially genetically determined as parietal lobe alterations were seen in monozygotic twins with SZ but not in their nonaffected twins [101]. There are studies indicating parietal structural alterations in the early phase of psychosis [99]. However, a reduction in the parietal volume was not found in the UHR sample from Melbourne [98]. This inconsistency could be explained by major methodological differences of both studies: the study by Borgwardt et al. spanned a time period of 3-4 years and the analysed MRI slices were 1 mm thick, whereas the Melbourne group observed their sample over 1 year and acquired MRI slices that were 3 mm thick [98, 99]. In a recent UHR study from Melbourne applying voxel-based morphometry methodology in 1.5 mm thick MRI slices, reductions in frontal and parietal cortices were detected in the UHR group who later converted to a psychotic disorder compared to those who did not convert [24]. 

The neurodevelopmental hypothesis suggests that early insults in pregnancy and infancy combined with genetic factors render the brain vulnerable for later development of SZ [108]. However, symptoms usually occur many years after the implied first damage [100, 109], hence a second insult was proposed to explain for the long delay [100]. Here, we want to propose that in a proportion of patients subsequently developing SZ, structural and functional alterations may start in parietal lobes progressing to frontal regions. The “parietal type” of SZ development may clinically present initially with working memory deficits [50, 56, 58, 60] and disturbed self-conceptualization [1, 84]. The “parietal type” may be more relevant for early onset forms of psychotic disorders in adolescents, with COS being the most extreme variant. In this model, genetic influences would play a more prominent role. Additional causative factors, yet to be identified, interacting with brain maturational processes in late adolescence and early adulthood may be decisive in the transition to a full-blown psychotic disorder [110]. Such an approach may enable us to characterise subtypes of SZ based on structural and functional brain processes and not merely on pure phenomenology. 

The functional interdependency of the parietal and frontal cortices was revealed in GHR subjects with isolated psychotic symptoms. They displayed compensatory activation in the left inferior parietal lobule while performing the Hayling sentence completion task [40]. Additionally, Quintana et al. reported parietal cortex activation in schizophrenics as compensatory mechanism for prefrontal cortex dysfunction while performing a working memory task [58]. However, Schneider et al. described parietal hypoactivation combined with hyperfrontality in first-episode schizophrenia patients with poorer performance [60]. In contrast to the study by Schneider and colleagues, in the studies conducted by Whalley and Quintana no statistical differences in performance accuracy were found between patients and controls. The hyperactivation of the PL may compensate for frontal hypoactivity, then the network between the frontal and parietal cortices is sufficient; hence tasks can be performed adequately. But in case of a disconnection in the frontoparietal network [50], the PL is not recruited to support the frontal lobe. This could lead to an inadequate hyperactivation of the frontal lobe leading to poor performance. This hypothesis warrants confirmation in carefully planned studies. 

Finally, some voxel-based morphometry studies have not found PL volume reductions in FEP [61, 111] and chronic SZ [108, 112]. More research is required to explore parietal lobe functions and volumes across different stages of SZ. Whether the pattern of parietal lobe changes is suitable for identifying a more homogeneous subgroup of patients with emerging SZ remains to be determined. Further longitudinal data are necessary from the earliest stages of SZ, particularly in prepsychotic individuals, to resolve this issue. A better understanding of the time course of structural and functional brain changes across different stages of psychotic disorders [109] will finally help us to distinguish between those individuals at incipient risk for a major mental illness and those with merely a transient crisis in life.

## Figures and Tables

**Table 1:** Studies reporting lesions in parietal lobe.

Investigator	Lesion site	Subjects	Affected function/impairment	Conclusion	Type of study
Danckert et al. 2002 [14]	Right PL	1 patient with visual neglect	Imagined movements did not show the same speed-accuracy trade-off observed for actual movements	Right parietal lobe may be important in the generation of internal models of motor movements	Case report

Doricchi and Tomaiuolo 2004 [15]	Right hemispheric damage	21 pts with neglect 10 ctrls	Neglect	Maximal overlap in supramarginal gyrus and superior longitudinal fasciculus; decisive role of parieto-frontal disconnection in neglect	Case series

Haaland et al. 2000 [16]	Left PL	41 pts with damage in left hemisphere	Ideomotor apraxia	Regions in the left hemisphere important for goal directed movements	Case series

Markowitsch et al. 1999 [17]	Left angular gyrus lesion	1 patient	Auditory working memory dysfunction	Calculation tasks were not affected	Case report

Paterson and Zangwill 1944 [18]	Penetrating head wound affecting the angular gyrus	1 patient		Neglect	Case report

Rosler et al. 1997 [19]	Ischemic lesion in the territory of the right middle cerebral artery	31 pts with right- or left- sided infarctions 31 ctrls	Facial recognition ability in pts with right-sided lesions was lessened compared with ctrls and with pts with left-sided infarctions	Graded impairment in patients with right middle cerebral artery infarcts	Cross-sectional case-control study

Rossetti et al. 2005 [20]	*First case*: bilateral parietal damage *Second case*: bilateral posterior parietal and upper and lateral occipital cortico-subcortical regions bilaterally through ischemic stroke	2 pts	*First case*: visual disorientation, simultanagnosia, and severe optic ataxia *Second case*: bilateral optic ataxia	Optic ataxia patients are impaired for immediate visuomotor processing but improve when required to delay before responding.	Case series

Sirigu et al. 1999 [21]	Left parietal cortex damage	3 pts with apraxia 6 ctrls with apraxia 2 nonapraxic neurological ctrs	pts were impaired in recognition of the viewed hand as the examiner's when it performed movements similar to their own movement	Parietal cortex is important for the perception of own movements as self-generated	Cross-sectional

PL: parietal lobe; pts: patients; ctrls: healthy controls.

**Table 2:** Recent structural magnetic resonance imaging (MRI) and computerized tomography (CT) studies on psychosis and schizophrenia with relevance to the parietal lobe.

Investigators	Subject groups	Average age of first scan (years)	Average years of followup	Image slice thickness	methods of analysis	Brain regions showing significant change in patients	Correlations between brain changes and clinical variables
Buchanan et al. 2004 [22]	44 csz 34 ctrls	39 34	0	1.5 mm	ROI manual tracing	Smaller inferior prefrontal region vol. and reversal of the normal asymmetry of the inferior parietal cortex	

Cannon et al. 2002 [23]	20 MZ discordant pairs 20 DZ discordant pairs 20 MZ ctrls 20 DZ ctrls	48 49 48 47	0	1.2 mm	Three-dimensional cortical maps	Between patients and their MZ cotwins reduced grey matter in the superior *parietal lobule*, dorsolateral prefrontal cortex, Broca's area, premotor cortex and frontal eye fields, superior temporal gyrus.	Disease-related deficits in grey matter were correlated with measures of symptom severity and cognitive dysfunction

Dazzan et al. 2011 [24]	102 UHR	20	1	1.5 mm	VBM	Reductions in the frontal cortex in subjects who developed psychosis and the subgroup that subsequently developed SZ also showed smaller volumes in the *parietal cortex *	

Dubb et al. 2005 [25]	46 csz 92 ctrls	29 31	0	1.0 mm	VBM	Reduced vol. of the parietal and frontal lobe	

Foong et al. 2001[26]	25 csz 30 ctrls	37 35	0	5.0 mm	MTI	Frontal and temporal vol. reductions	Bilateral parieto-occipital cortex and genu of corpus callosum vol. reductions were associated with severity of negative symptoms in sz

Frederikse et al. 2000 [27]	15 male scz 15 female csz 15 male ctrls 15 female ctrls	39 40 39 38	0	1.5 mm	ROI of the inferior parietal lobule	Male csz had a reversal of the normal left greater than right male asymmetry and smaller left inferior parietal lobule grey matter vol. female csz did not differ from female ctrls	

Hubl et al. 2004 [28]	13 csz with to auditory hallucinations 13 csz without auditory hallucinations 13 ctrls	33 31 32	0	5.0 mm	DTI	pts with hallucinations had higher white matter directionality in the lateral parts of the temporoparietal section of the arcuate fasciculus and in parts of the anterior corpus callosum	Alterations of white matter fiber tracts in pts with frequent hallucinations lead to abnormal coactivation in regions related to the acoustical processing

Job et al. 2005 [29]	65 GHR 19 ctrls	21 21	2	1.9 mm	VBM	GHR with *right parietal grey matter*, temporal grey matter, right frontal grey matter reductions	GHR with psychotic symptoms and converters showed a different spatial pattern of reductions

Jung et al. 2009 [30]	29 UHR 31 SZ 29 ctrls	22 24 23	0	voxel size 0.45 × 0.45 × 0.9 mm	VBM	UHR: cortical thinning in prefrontal cortex, anterior cingulate cortex, inferior parietal cortex, parahippocampal cortex, and superior temporal gyrus	Cortical thinning was more pronounced in SZ compared with UHR and ctrls

Kubicki et al. 2001 [31]	16 FE 16 affective psychosis 18 ctrls	26 23 24	0	1.5 mm	VBM	FE reduced volume of right inferior parietal lobule, right dorsolateral prefrontal cortex, left and right anterior cingulate gyrus, left and right insula	

Kyriakopoulos et al. 2009 [32]	17 adolescent-onset SZ 17 adolescent ctrls 17 adult-onset SZ 17 adult ctrls	17 16 24 24	0	2.5 mm	DTI	Individuals with adolescent onset SZ show fractional anisotropy decrease in parietal regionsindividuals with adult onset show additionally in frontal, temporal, and cerebellar regions	White matter abnormalities in SZ may depend on developmental stage at the time of illness onset

Minatogawa-Chang et al. 2009 [33]	88 FEP 86 ctrls	29 31	0	voxel size 2 × 2 × 2 mm^3^	MRI + controlled oral word association test + forward and backward digit span tests	Volume abnormalities in frontal and temporoparietal cortices	Cognitive deficits directly related to brain volume abnormalities in frontal and temporoparietal cortices in FEP subjects

Mitelman et al. 2009 [34]	17 csz with good outcome 17 csz with poor outcome 13 ctrls	37 47 42	4	1.2 mm	DTI	At baseline, csz had smaller frontal, temporal, and parietal gray matter volumes than ctrls	Grey matter volumes in poor-outcome patients decline more rapidly than in patients with good outcome

Narr et al. 2005 [35]	72 FE 78 ctrls	25 27	0	1.5 mm	MRI	Regional grey matter thinning in frontal, temporal and *parietal* heteromodal association cortices bilaterally	

Nierenberg et al. 2005 [36]	14 FE 14 ctrls	18–55	0	1.5 mm	ROI	Smaller left angular gyrus vol.	

Niznikiewicz et al. 2000 [37]	15 male right-handed csz 15 male right-handed ctrls	20–55	0	1.5 mm	ROI	Showed a reversed asymmetry in the inferior parietal lobule that was mainly seen in the angular gyrus	

Rowland et al. 2008 [38]	10 csz with neg. symptoms 10 csz without neg. symptoms 11 ctrls	46 40 37	0	2.2 mm	DTI	Reduced FA in the superior longitudinal fasciculus connecting parietal with frontal lobe	Support for altered frontal-parietal network in deficit SZ

Schultz et al. 2009 [39]	54 FE 54 ctrls	26 27	0	1 mm	MRI	Cortical thinning in: dorsolateral and frontopolar cortices, anterior cingulate cortex, superior temporal cortices, and superior parietal lobe	Widespread reduction of cortical thickness, mostly in heteromodal cortices of frontotemporal networks

Sun 2003 [40]	23 UHR-N 12 UHR-P	20 20	1	1.5 mm	MRI	*UHR-P *: reduction in dorsolateral prefrontal cortex and orbitofrontal cortex	High-risk psychosis subjects showed orbitofrontal cortex reduction compared to FE

Sun et al. 2005 [41]	23 FEP (16 FES) 11 csz 28 ctrls	22 33 26	2	1.5 mm	MRI	*FE versus ctrls*: whole brain, left (trend), and right motor-premotor, *left and right parietal*, left and right dorsal prefrontal *csz versus FE: *left and right dorsal prefrontal	FE brain surface retraction was similar to that of ctrls but significantly accelerated

Thompson et al. 2001 [42]	12 SZ 12 ctrls	14 14	4.6	1.2 mm	MRI	*Earliest scans: *deficits in parietal brain regions *Latest scans: *included dorsolateral prefrontal cortex and superior temporal gyri	Change patterns correlated with psychotic symptom severity

Whitford et al. 2005 [43]	31 FE 30 ctrls	19 19	0	1.5 mm	VBM	left prefrontal cortex, *left parietal* and temporal cortex, right cerebellum, *right parietal*, frontal, and cortex reductions	Reality distortion syndrome score correlates with grey matter reduction in FE

Whitford et al. 2006 [44]	41 FES followup: 25 FES 47 ctrls followup: 26 ctrls	19 19	2.6 2.4	1.5 mm	VBM	*Baseline: *grey matter reductions in the frontal, *parietal*, and temporal cortices and cerebellum *Followup interval: *especially in the *parietal* and temporal cortices further reductions	

Narr et al. 2001 [35]	48 csz 48 ctrls	33 32	0	0.9–1.4 mm	VBM	Left-dominant frontal, temporal, and insular grey matter reductions	Global assessment of functioning score correlated with grey matter vol. in the left inferior frontal and inferior parietal lobe

Zhou et al. 2006 [45]	53 csz 25 schizotypal disorder pts 59 ctrls	25 26 24	0	1 mm	ROI	csz: reduction in parietal lobe schizotypal subjects: postcentral gyrus volume reductions	

csz: chronic schizophrenic patients; ctrls: healthy controls; ROI: region of interest; vol.: volume; MZ: monozygotic twins; DZ: dizygotic twins; VBM: Voxel-Based Morphometry; MTI: magnetization transfer imaging; SZ: schizophrenia; DTI: diffusion tensor imaging; FE: first episode schizophrenia; FEP: first episode psychosis; FA: fractional anisotropy; GHR: subjects at genetically high risk; UHR: ultra-high-risk patients; UHR-P: ultra-high-risk subjects who became psychotic; UHR-N: ultra-high-risk subjects who did not became psychotic, VBM: voxel-based morphometry.

**Table 3:** Recent functional magnetic resonance imaging (MRI) and positron emission tomography (PET) studies on psychosis and schizophrenia with relevance to the parietal lobe.

Investigator	Subject groups	Average age	Paradigm	Tested brain function	Method of analysis	Main findings in patients compared with ctls	Conclusion
Arce et al. 2006 [46]	17 csz 17 ctrls	41 40	Visual Go/Nogo task with matched performance accuracy between csz and ctrls	Inhibition and cue processing	fMRI	*During cued inhibition*: greater activation in the left precuneus and left superior temporal gyrus *During inhibition*: less ACC and DLPFC activation *Implicit cue trials*: greater inferior frontal gyrus activation	Csz have difficulties with inhibition and clue processing

Braus et al. 2006 [47]	11 FE 11 ctrls	25 29	Simultaneous presentation of acoustical and optical inputs	Basic sensory input circuits	fMRI	Less activation of the PL, right thalamus, the right prefrontal cortex	Already at disease onset deficits in information processing are existing

Broome et al. 2009 [48]	17 UHR 10 FEP 15 ctrls	24 26 25	Verbal fluency task and an N-back	WM	fMRI	Activation pattern in UHR was during the N-back task different in dorsolateral prefrontal and parietal cortex compared to ctrls	The level of regional activation in the UHR group was intermediate between that in the FE group and ctrls

Franck et al. 2002 [49]	87 csz	31	Instructed to relax and not perform any tasks	Random episodic silent thought (REST)	PET	Schneiderian score positively correlated with rCBF in right superior parietal cortex and negatively correlated with rCBF in left posterior cingulate gyrus and in left lingual gyrus	Findings support hypothesis that cerebral pattern of activation is linked to symptoms of SZ

Henseler et al. 2010 [50]	12 csz 12 ctrls	33 32	Verbal item-recognition task and a visuospatial item-recognition task	WM	fMRI	SZ showed reduced connectivity of the prefrontal cortex with the intraparietal cortex and the hippocampus	Altered prefronto-hippocampal and parieto-occipital connectivity was found to be associated with higher positive symptoms

Hugdahl et al. 2004 [51]	csz depressed pts ctrls	32 33 31	First task: pressing a response button whenever a specific number was seen Second task: adding two consecutive numbers	Vigilance task Mental arithmetic task	fMRI	Less activation in prefrontal brain regions and greater parietal lobe activation relative compared to ctrls and patients with major depression.	In support double dissociation of parietal and frontal lobe activation between SZ and depression

Keedy et al. 2006 [52]	15 FE 24 ctrls	25 25	Eye movement tasks: visually guided saccade, smooth pursuit paradigms and oculomotor delayed response paradigm	Oculomotor function Spatial working memory	fMRI	Reduced activation in sensorimotor areas supporting eye movement control: parietal cortex, frontal eye fields, supplementary eye fields, and cingulated cortex	Generalized pattern of cortical dysfunction already present early SZ

Keshavan et al. 2002 [53]	4 GHR 4 ctrls	13 13	Memory-guided saccade task	Spatial working memory	fMRI	Decreased activation in the inferior parietal cortex and the DLPFC	Dysfunction of prefrontal and parietal regions in GHR

Kim et al. 2003 [54]	12 csz 12 ctrls	26 26	n-back sequential picture task	WM	PET	Dorsolateral prefrontal, ventrolateral prefrontal and bilateral inferior parietal region activation abnormalities	Indicating that during working memory tasks there could be a parietofrontal disconnection

Öngür et al. 2006 [55]	20 csz 17 ctrls	40 38	Discrimination of previously seen and new pairs of visual stimuli	Relational memory	fMRI	While discriminating novel pairs decreased activation of the right parietal cortex and the anterior cingulate cortex	Deficit of relational memory connected to dysfunctional activation of the parietal cortex and the hippocampus

Ojeda et al. 2002 [56]	11 csz drug naive 10 ctrls	28 26	Auditory stimulation task Counting tasks with or without auditory stimulation	Attention tasks	PET	Inadequate activation of parietal and frontal regions during performance of cognitively emanding tasks	Evidence of compensatory mechanisms in frontoparietal regions

Paulus et al. 2003 [57]	15 scz 15 ctrls	42 41	Two-choice prediction task	Decision making with different degrees of uncertainty	fMRI	PL less activated in decision making in situations with high uncertain outcome	Inadequate processing in situations of uncertainty in the posterior parietal cortex

Quintana et al. 2003 [58]	8 sz 8 ctrls		Anticipatory task Retention task	WM	fMRI	Anticipatory task: decreased PFC and increased PPC activation Retention task: increased PFC activation	PFC shows more hypoactivation than PPC is able to compensate

Sanz et al. 2009 [59]	13 csz no information about ctrls included	20	verbal capacity task	verbal WM	fMRI	Lower levels of activation in frontal lobe, PL in the left hemisphere	Dysfunctional activation during WM processing related to the severity of negative and disorganized symptoms

Schneider et al. 2007 [60]	48 FE 57 ctrls	31 31	2- and 0-back tasks	WM attention connected processes	fMRI	*Working memory*: parietal hypoactivations, combined with hyperfrontality in VLPFC Attention-connected processes: hypoactivations in the VLPFC, superior temporal cortex, thalamus	Dysfunctional cerebral network not able to cope with required activation for attention and WM tasks

Sweeney et al. 2003 [61]	8 csz with auditory hallucinations 8 ctrls	31 29	Fast versus slow covert articulation of a word at two self-paced rates	Processing inner speech	fMRI	Reduced activation in the right superior temporal, inferior parietal, and parahippocampal regions	SZ patients with auditory hallucinations have aberrant activation pattern of brain regions

Spence et al. 1997 [1]	7 d-scz 6 nd-scz 6 ctrls	Not stated	Performing movement task Second PET 4–6 weeks after first PET	Movement	PET	Increased right parietal and cingulate activation in csz with delusions of control but in d-scz with decreased passivity delusion in second scan hyperactivation of right parietal and cingulate remitted	Certain brain regions involved in generating delusions of passivity

Thermenos et al. 2005 [62]	14 SZ 22 ctrls	38 38	Visual letter 2-back task	WM	fMRI	Greater activation in the right medial frontal gyrus and left inferior parietal lobule/medial temporal gyrus region	Heteromodal association cortices show higher activation in SZ during performance of WoM task

Whalley et al. 2006 [63]	4 GHP-S 26 GHR-P 27 GHR-N 21 ctrls	23 26 27 27	Hayling sentence completion test	Word retrieval	fMRI and ROI	GHR-N showed increased activation of the PL and the anterior cingulate	PL and the lingual gyrus could be used to discriminate between converts and nonconverts

Whalley et al. 2004 [64]	21 GHR-P 48 GHR-N 21 ctrls	25 27 27	Part of Hayling sentence completion test	Verbal initiation	fMRI	GHR-P: increased activation in the left inferior parietal lobule GHR-N: less activity in medial prefrontal, thalamic and cerebellar regions	Soonest changes in patients with symptoms may be connected to hyperactivation in the parietal lobe

csz: patients with SZ; ctrls: healthy controls; fMRI: functional magnetic resonance imaging; ACC: anterior cingulate cortex; DLPFC: dorsolateral prefrontal cortex; FE: patients with first-episode schizophrenia; PL: parietal lobe; UHR: ultra-high risk patients with prodromal symptoms of schizophrenia; FEP: first episode psychosis; WM: working memory; PET: positron emission tomography; rCBF: regional cerebral blood flow; pts: patients; GHR: genetically defined high-risk for schizophrenia; PFC: prefrontal cortex; PPC: posterior parietal cortex; VLPC: ventrolateral prefrontal cortex; GHR-N: ultrahigh-risk subjects who remain nonpsychotic; GHR-P: ultra-high-risk subjects who became psychotic; GHP-S: ultra-high-risk subjects who became schizophrenic; nd-scz: schizophrenic patients without delusion of control; d-scz: schizophrenic patients with delusion of control; ROI: region of interest; SZ: schizophrenia.
